# Supplementary figures and images for: Coptidis alkaloids extracted from Coptis chinensis Franch attenuate IFN-γ-induced destruction of bone marrow cells
Source: PLoS One. 2020 Jul 24;15(7):e0236433. doi: 10.1371/journal.pone.0236433 (PMC7380622; doi:10.1371/journal.pone.0236433)

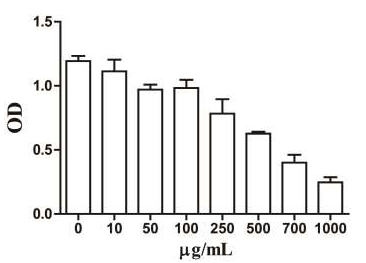

Supplement: S1 Fig — (TIFF) [file pone.0236433.s001.tiff]
