# Supplementary material for: Coptidis alkaloids extracted from Coptis chinensis Franch attenuate IFN-γ-induced destruction of bone marrow cells
Source: PLoS One. 2020 Jul 24;15(7):e0236433. doi: 10.1371/journal.pone.0236433 (PMC7380622; doi:10.1371/journal.pone.0236433)
Supplement: S1 File — (PDF) [file pone.0236433.s002.pdf]

Pictures of uncropped western blots

Figure 3. Interference in the protein expressions of key molecules in Fas apoptotic spathway in BMCs induced by IFN-γ after 12 h of Coptidis alkaloids treatment.

Caspase3:

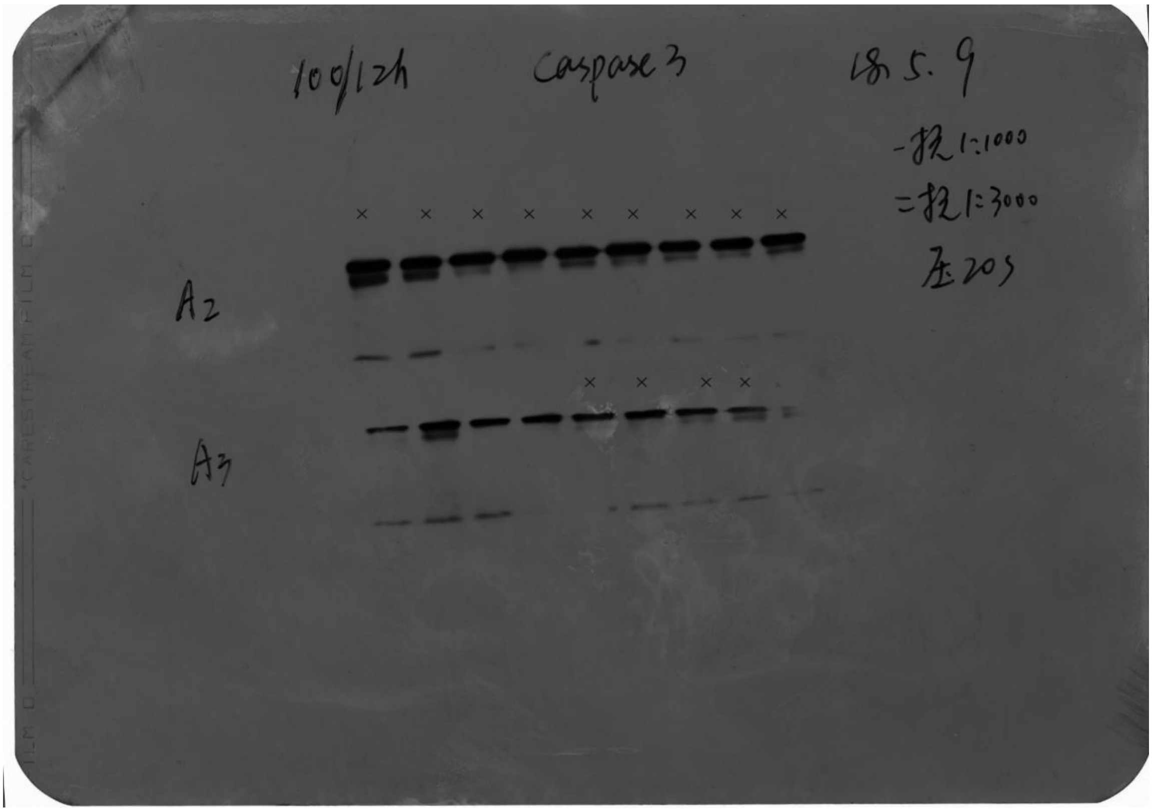

Cleaved caspase3:

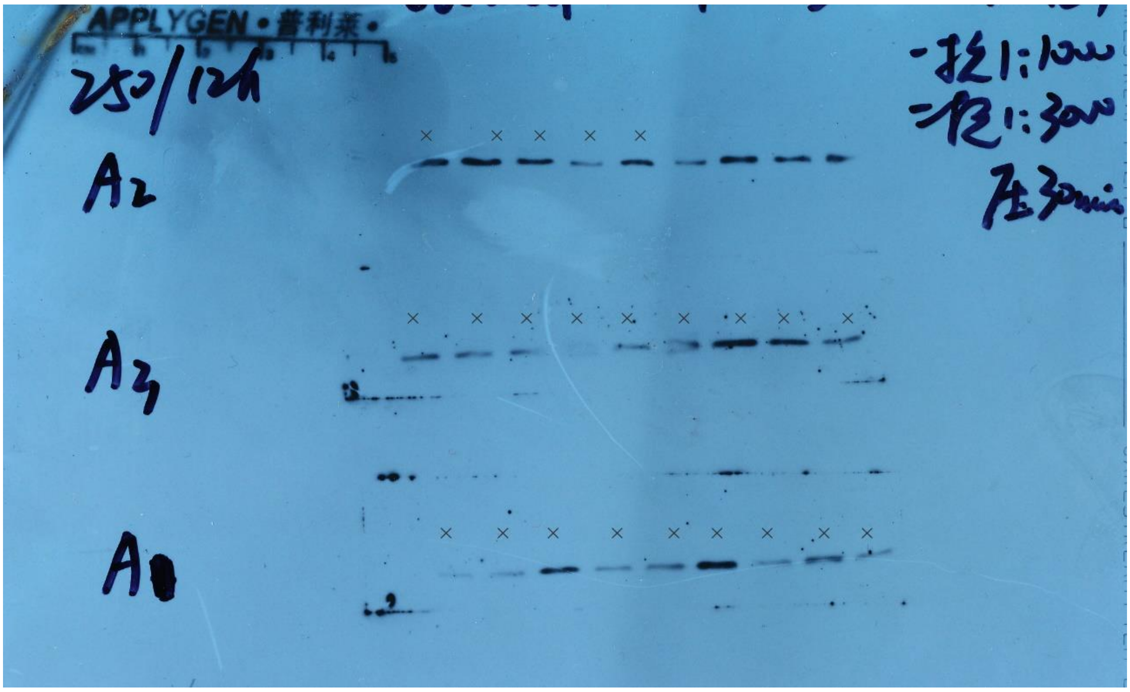

Caspase8:

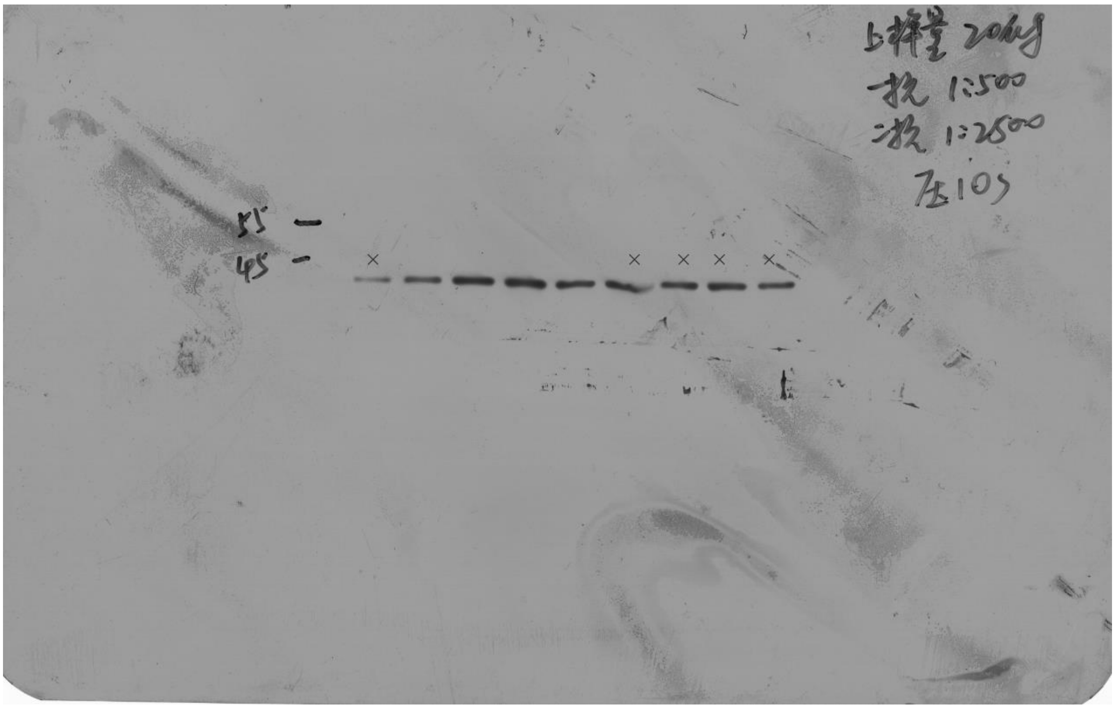

Cleaved caspase8:

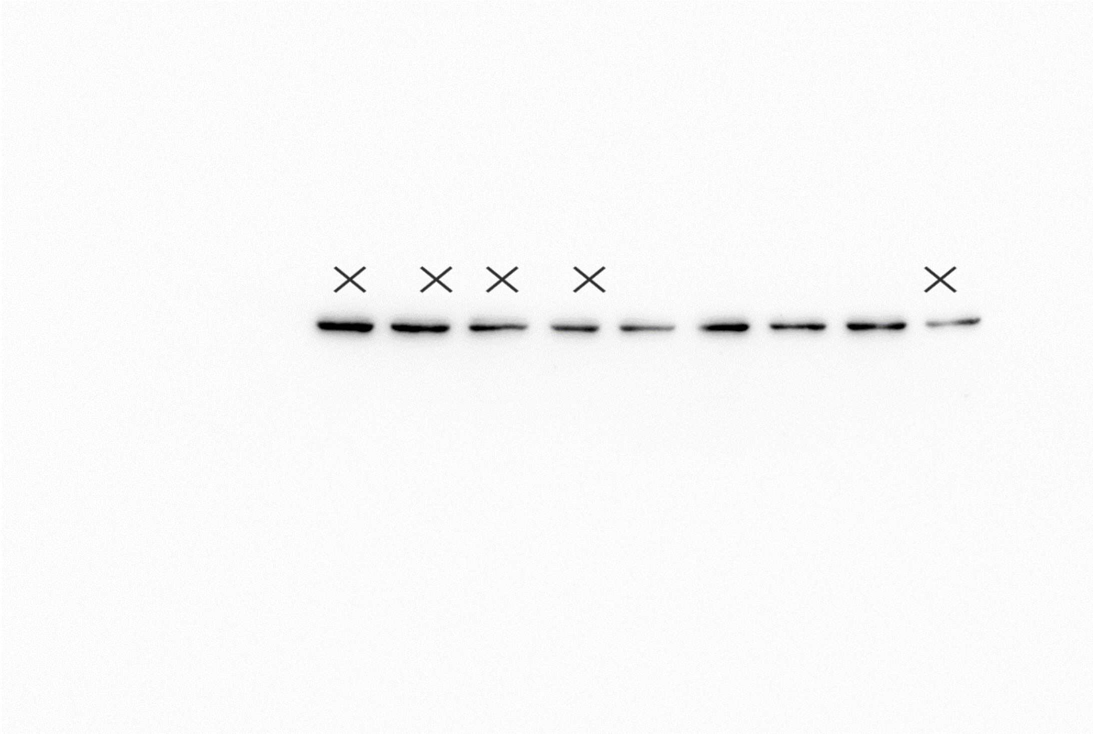

Fas:

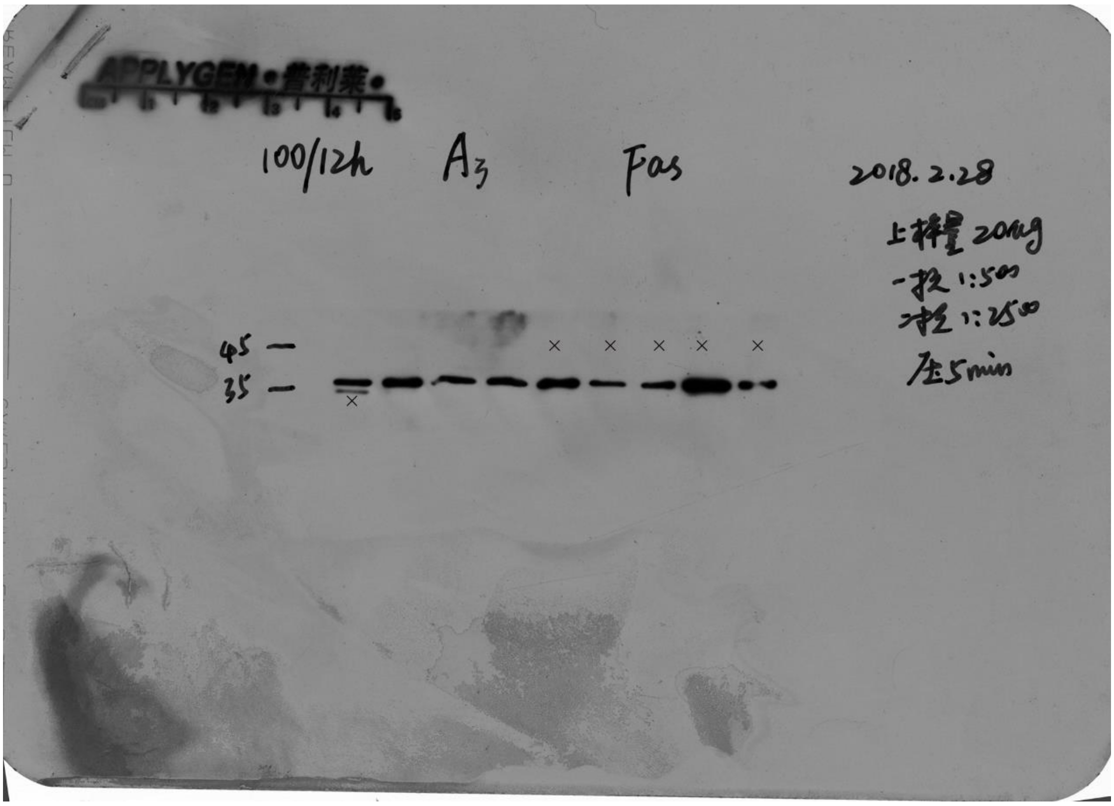

β-actin:

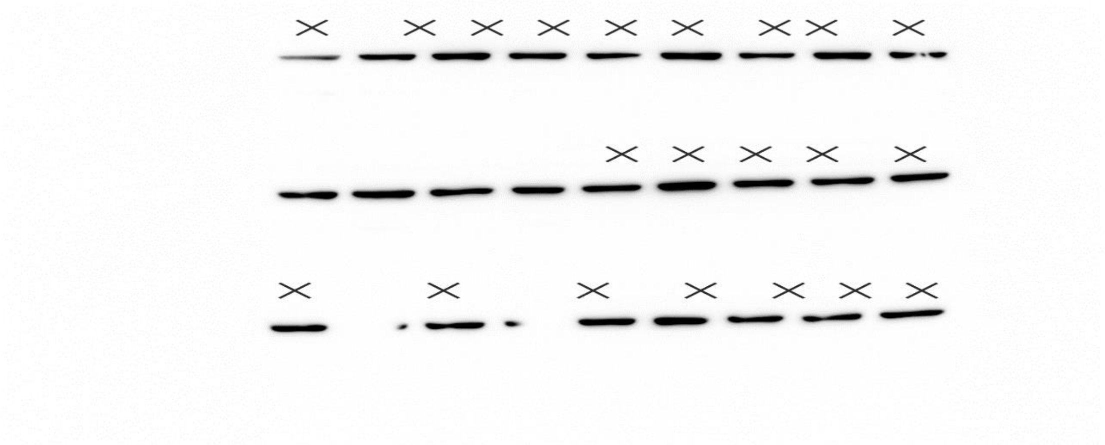

Figure 4. Interference in the protein expressions of key molecules in Fas apoptotic spathway in BMCs induced by IFN-γ after 24 h of Coptidis alkaloids treatment.

Caspase3:

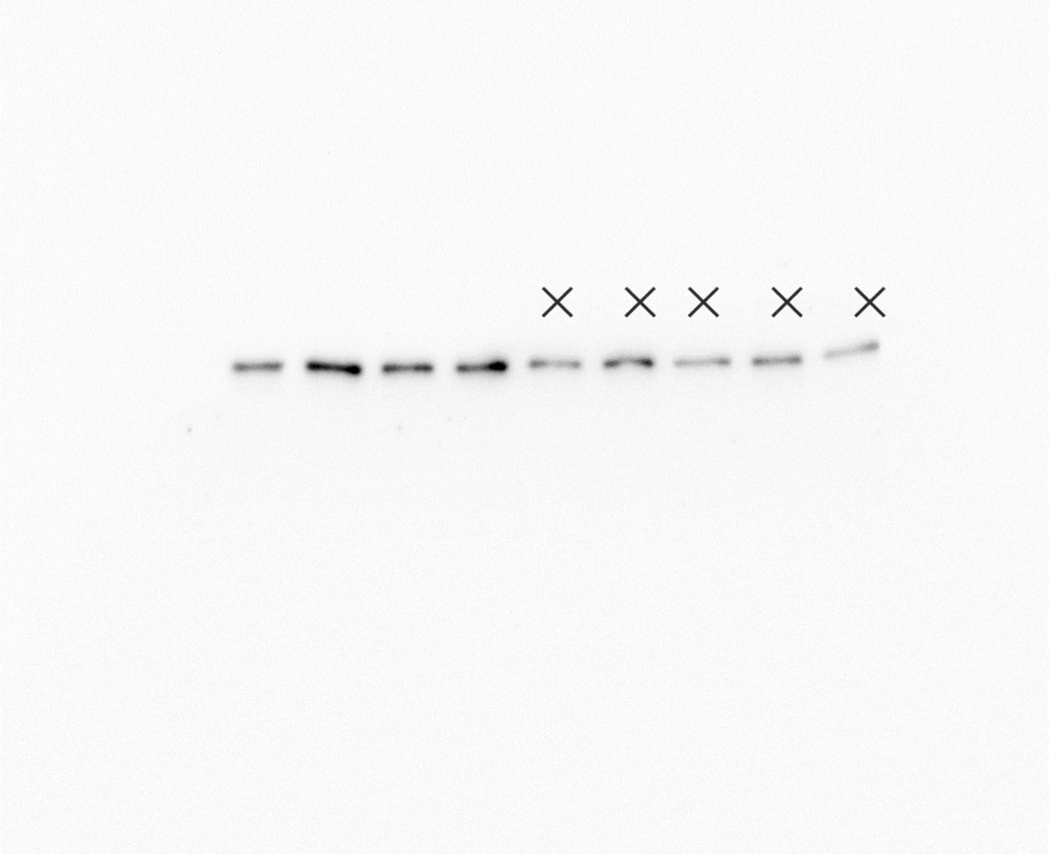

Cleaved caspase3:

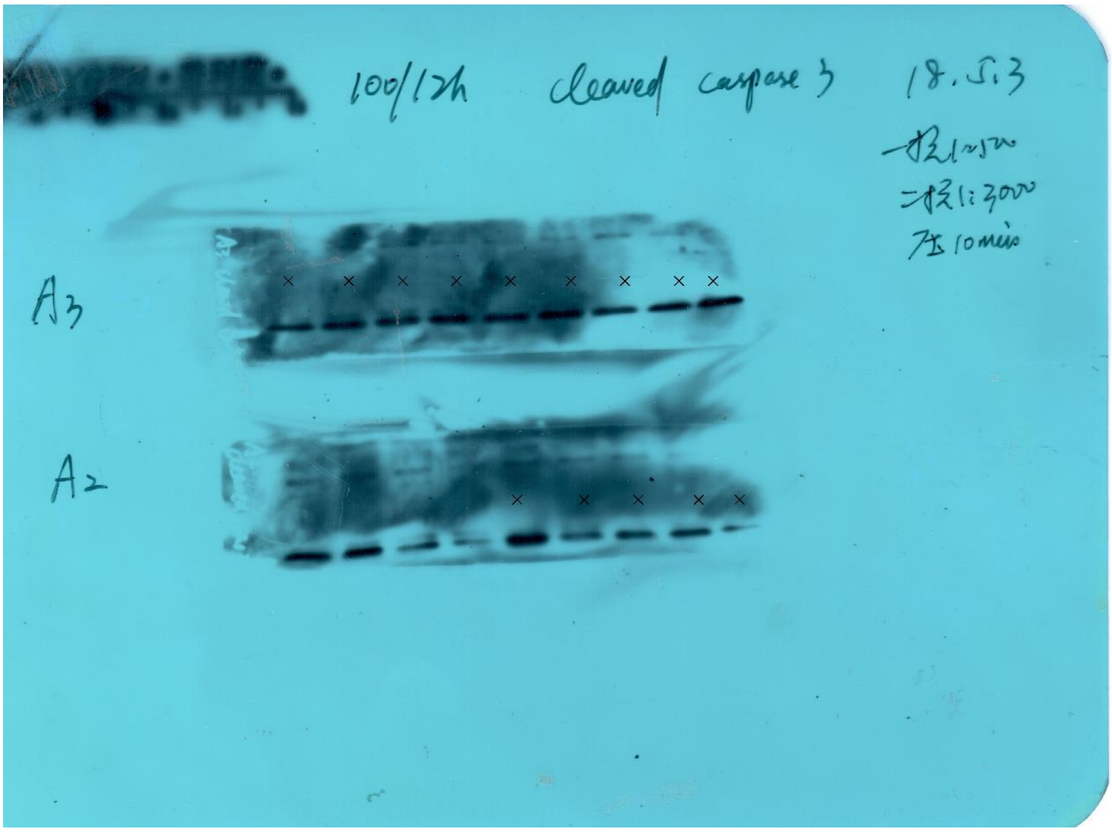

Caspase8:

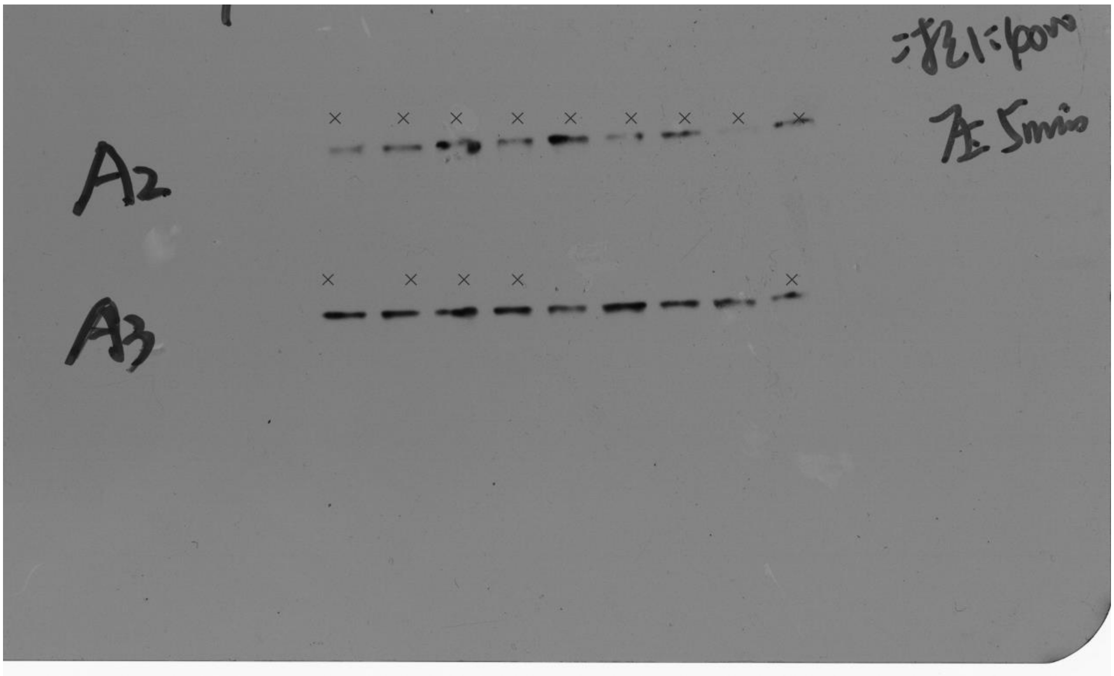

Cleaved caspase8:

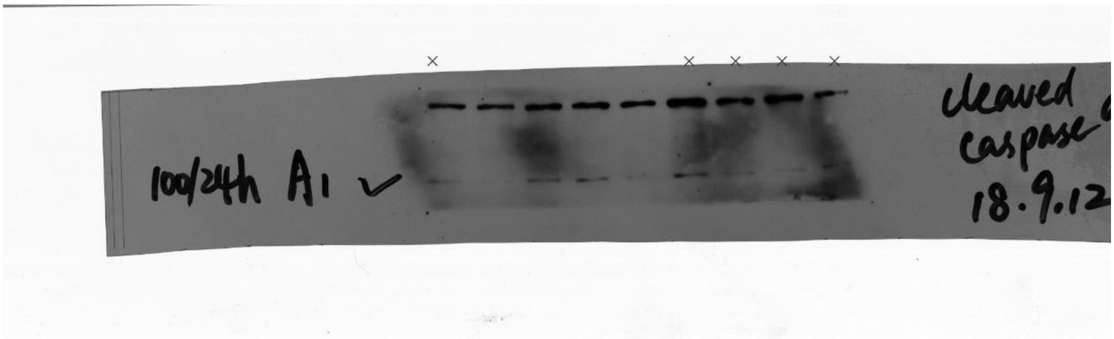

Fas:

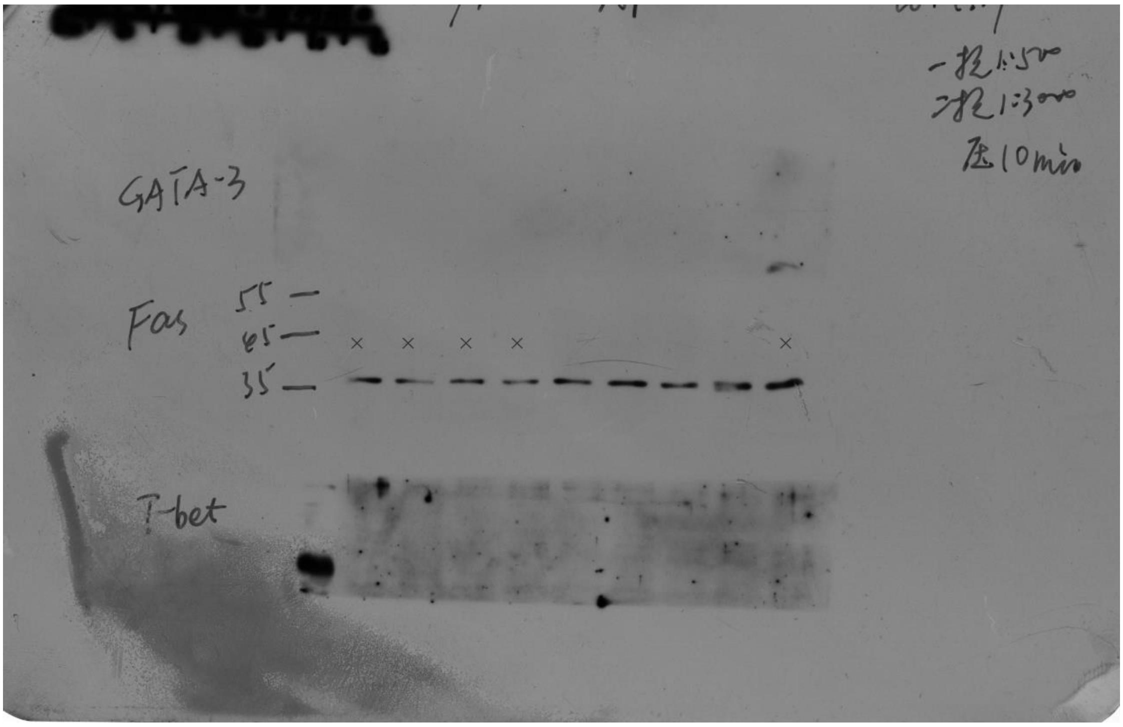

β-actin:

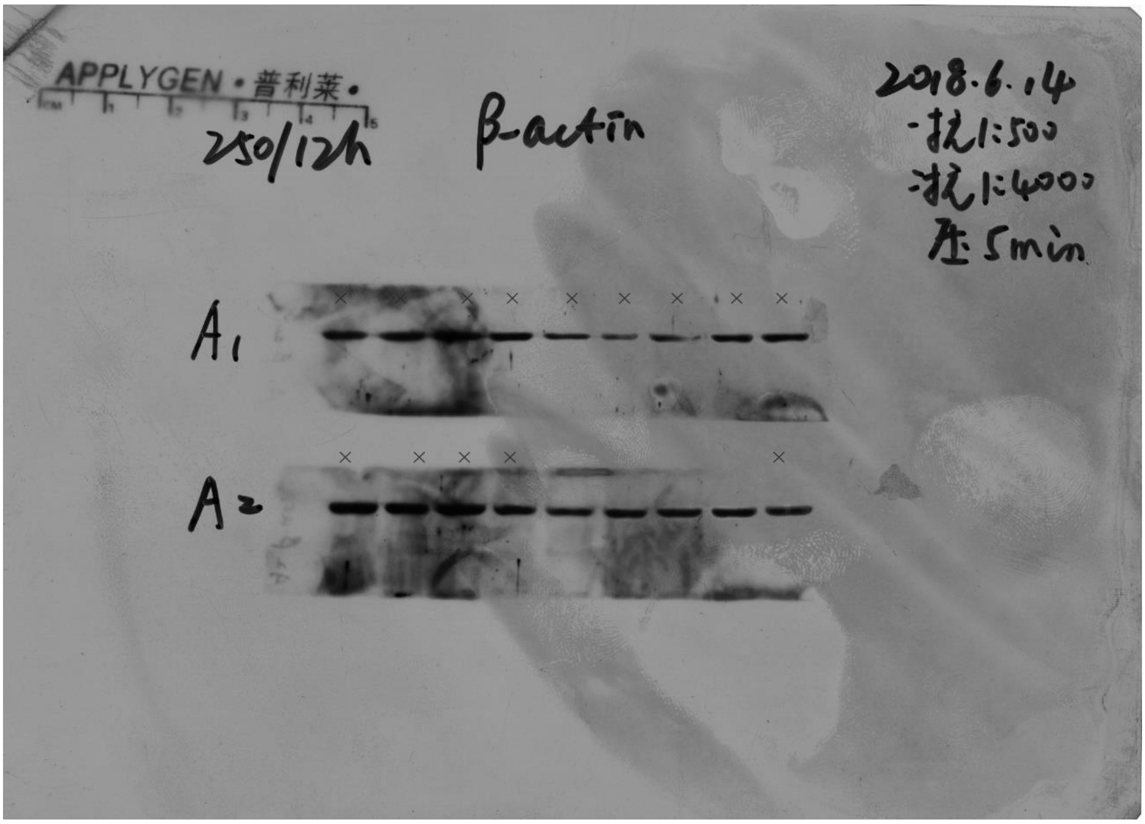

Figure 11. (A) The protein levels of key molecules in Jak/Stat signal pathway in BMCs

induced by IFN-γ after 12 h of treatment

Stat-1:

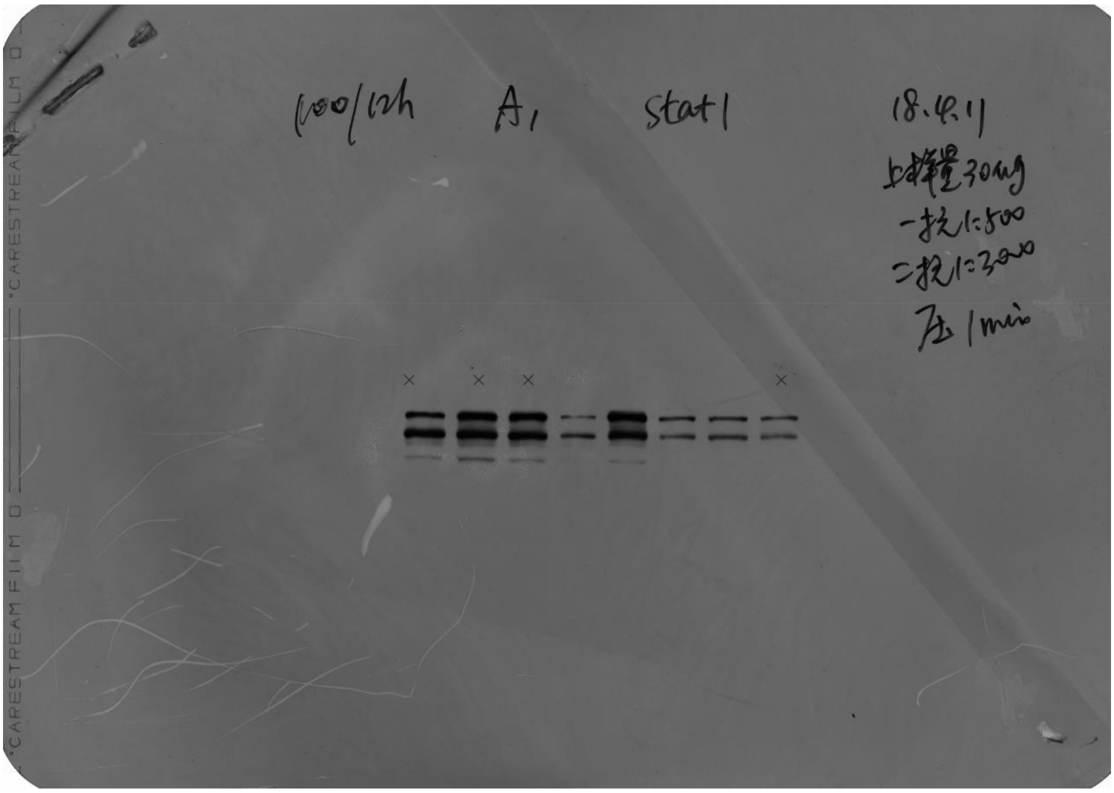

p-Stat-1:

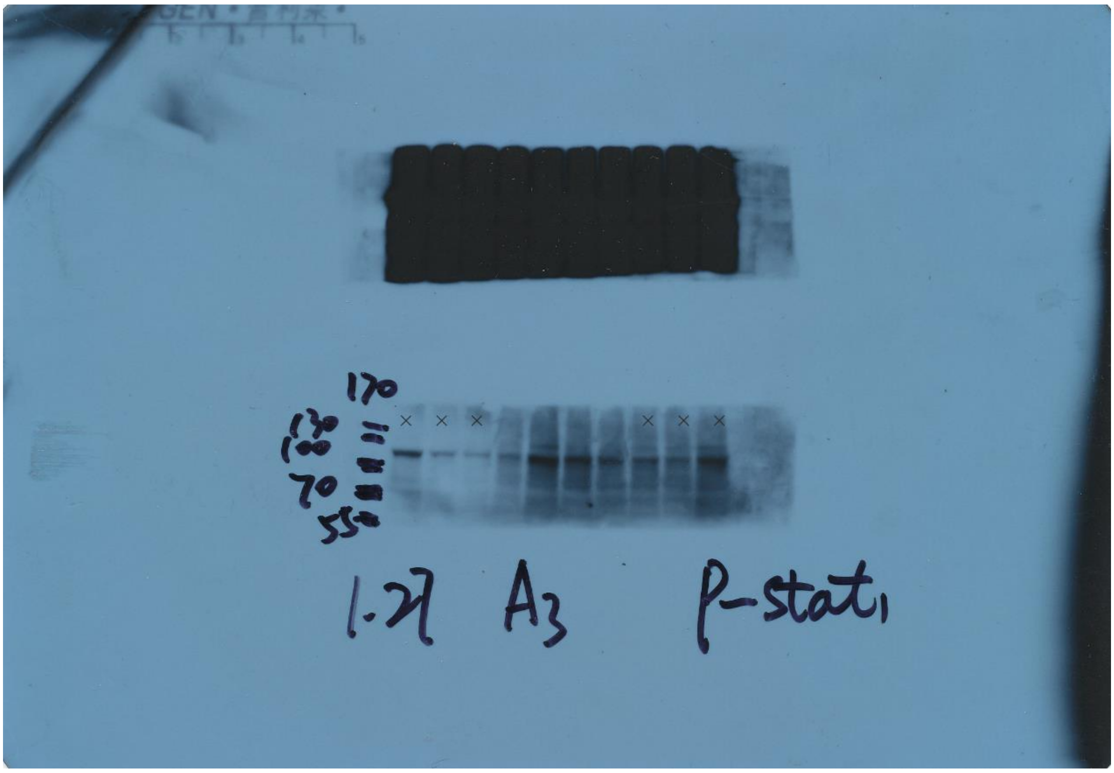

Stat-3:

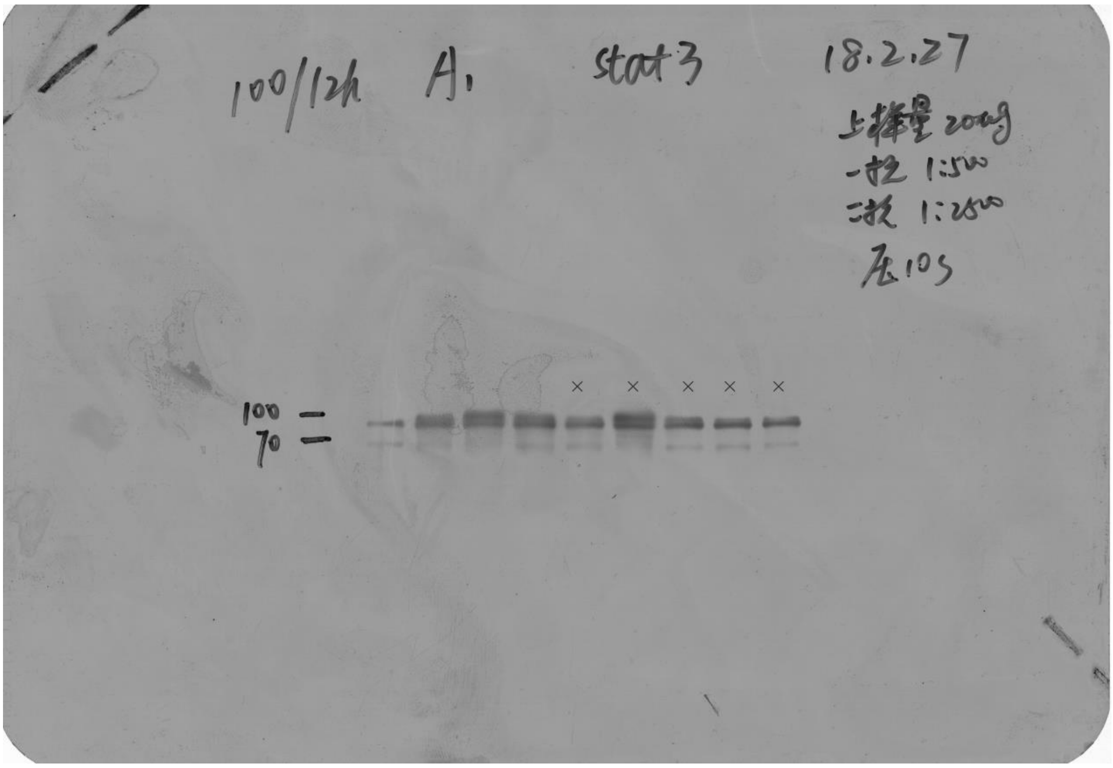

p- Stat-3:

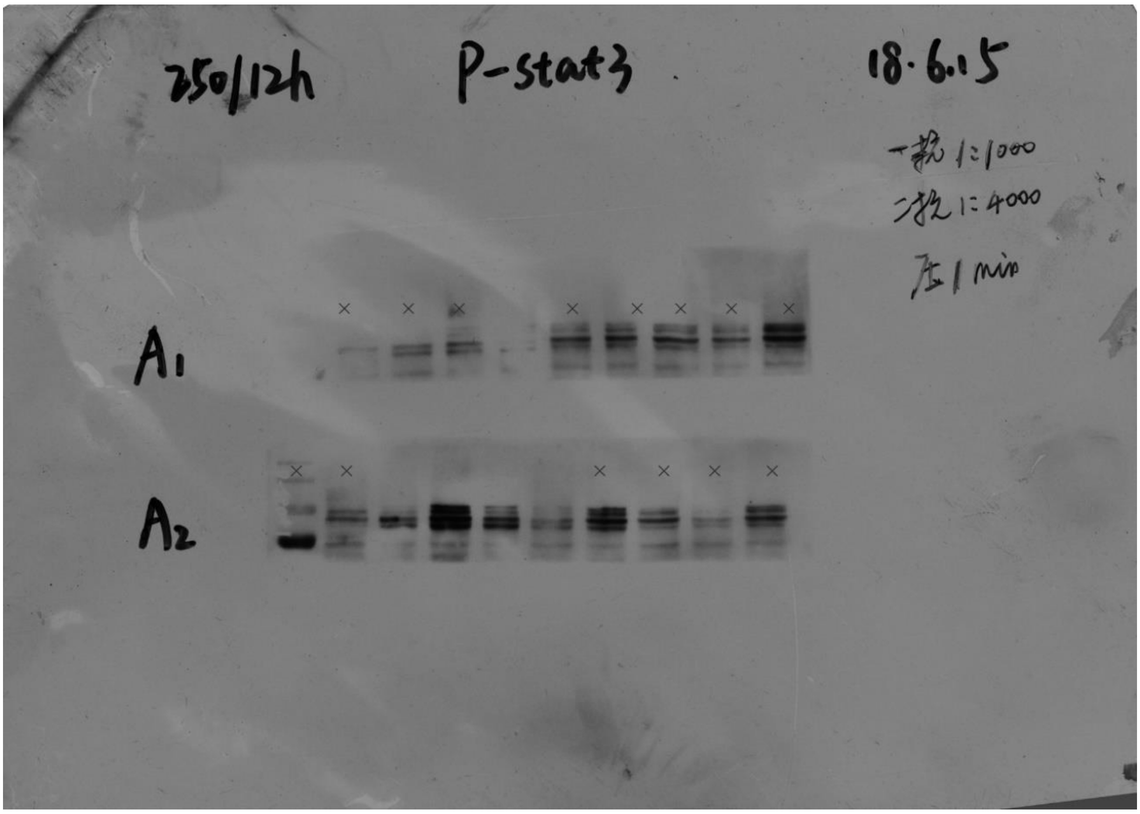

β-actin:

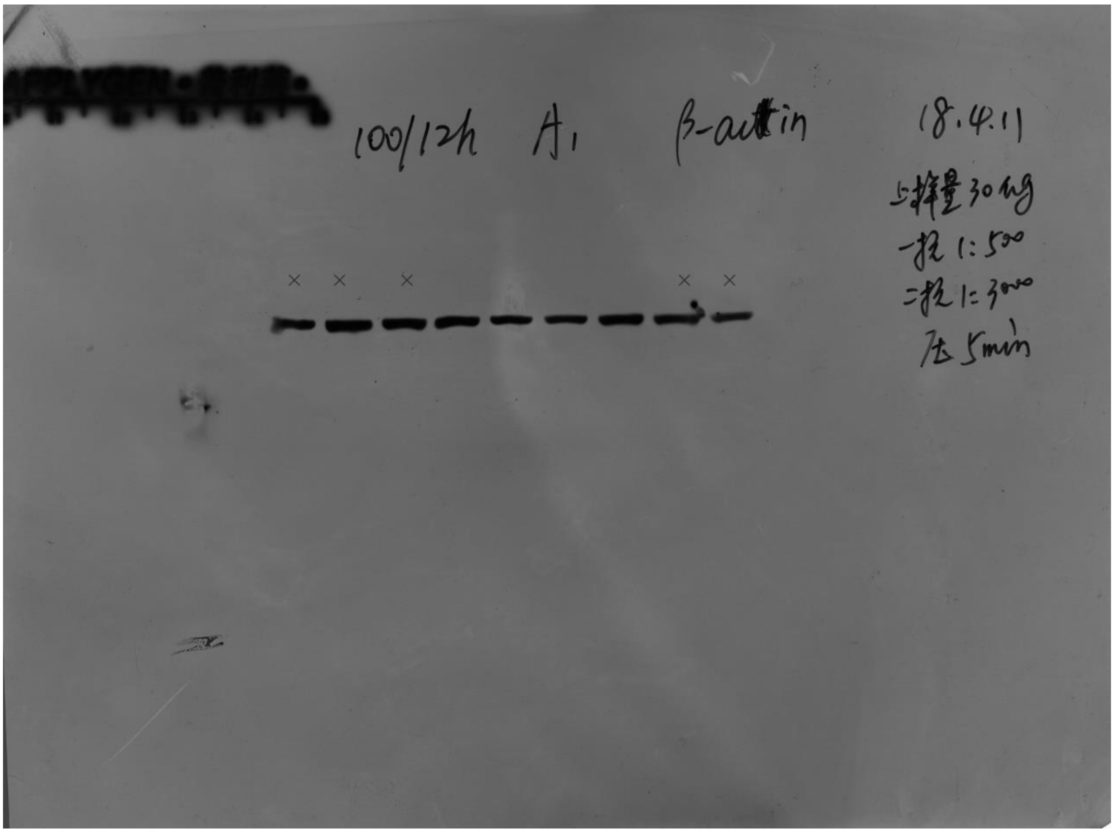

Figure 11. (B) The protein levels of key molecules in Jak/Stat signal pathway in BMCs induced by IFN-γ after 24 h of treatment

Stat-1:

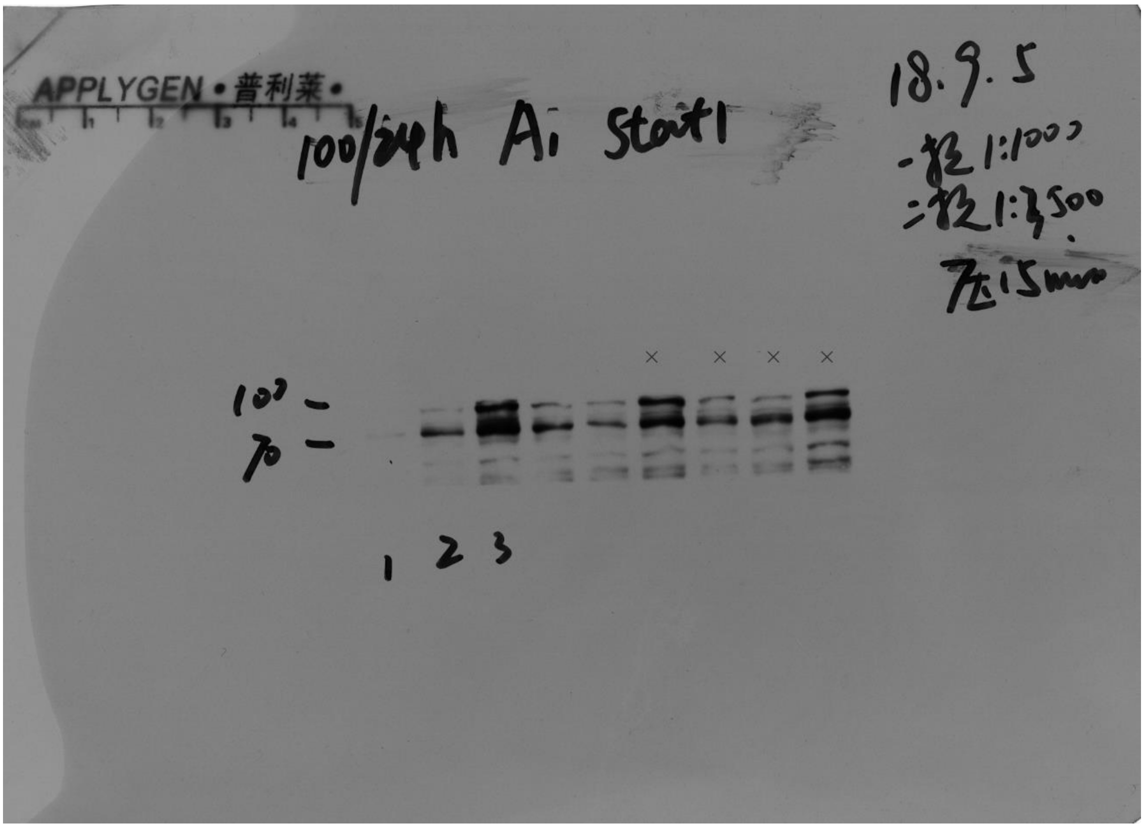

p-Stat-1:

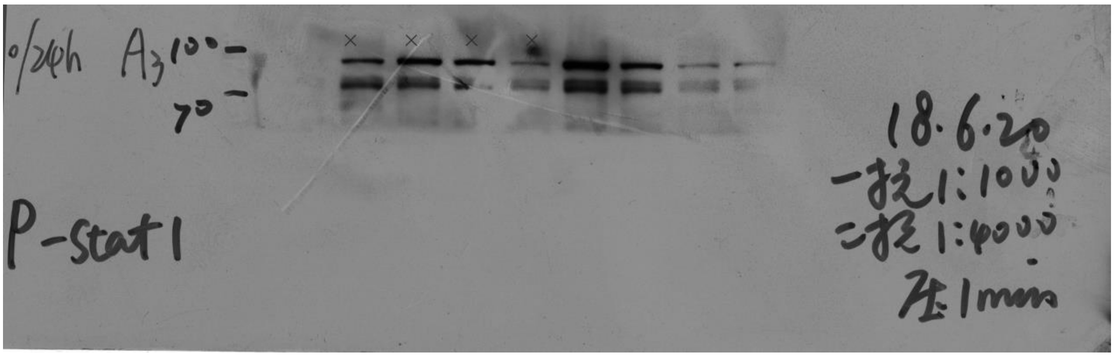

Stat-3:

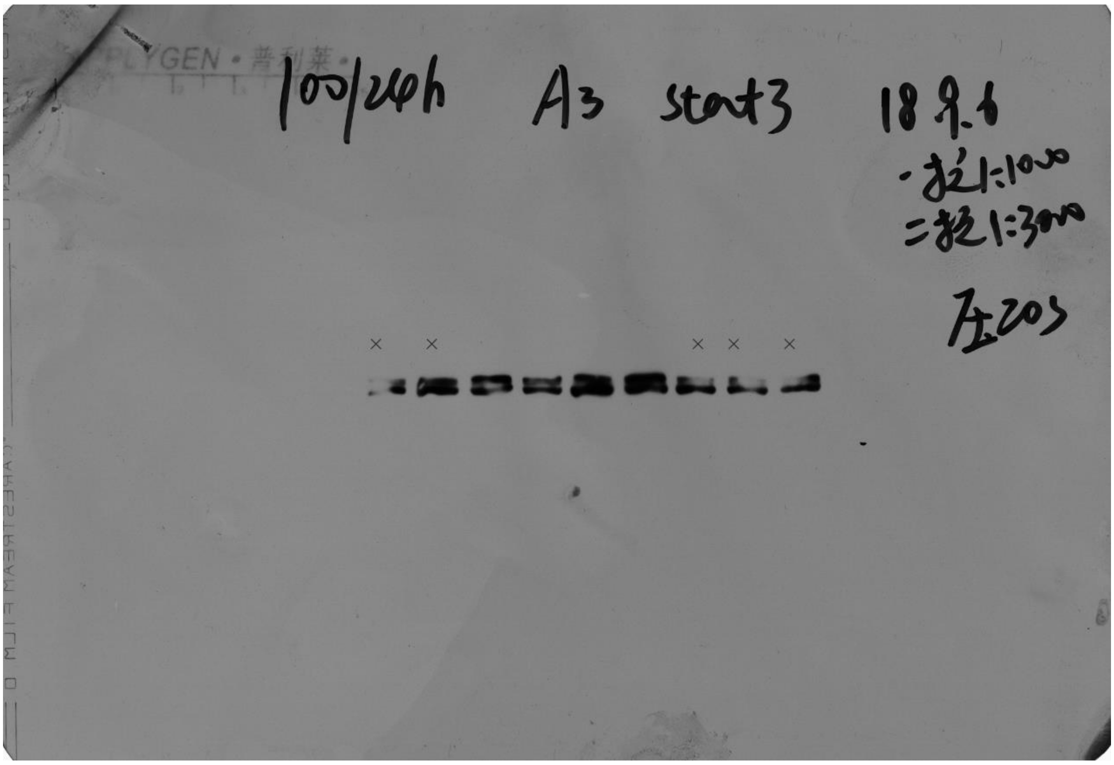

p-Stat-3:

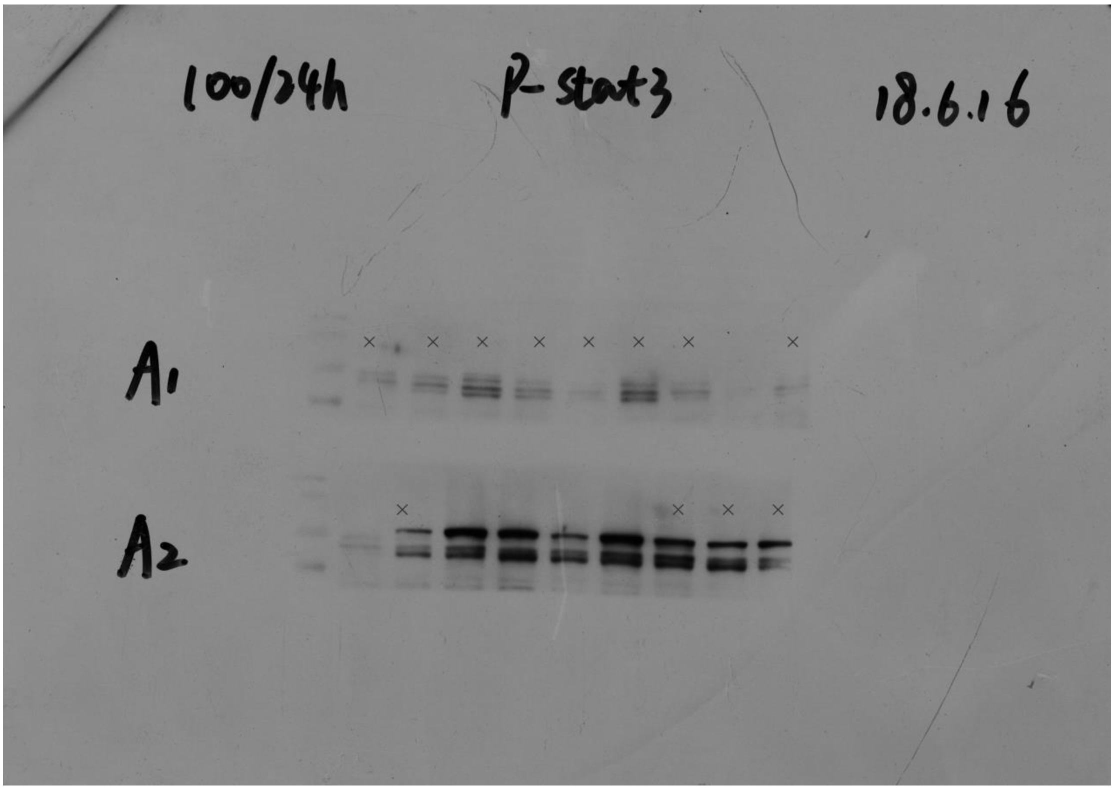

β-actin:

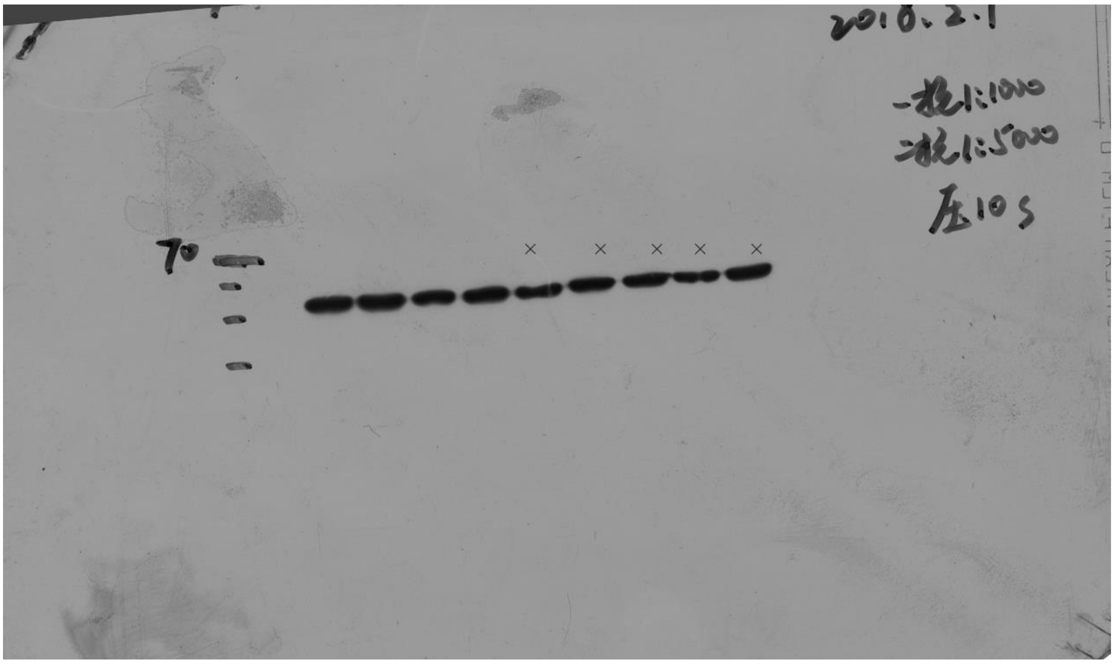
2018.2.1
一抗 1:1000
二抗 1:5000
压 10 s
70
